# Supplementary material for: The burden and trends of child and maternal malnutrition across the regions in Ethiopia, 1990–2019: The Global Burden of Disease Study 2019
Source: PLOS Glob Public Health. 2024 Jul 16;4(7):e0002640. doi: 10.1371/journal.pgph.0002640 (PMC11251601; doi:10.1371/journal.pgph.0002640)
Supplement: Attachment — Submitted filename: Response To Reviewers.docx [file pgph.0002640.s001.docx]

**Response To Reviewers**

Editor comments

We noticed you have some minor occurrence of overlapping text with the following previous publication(s), which needs to be addressed:

Response : Thank you for the comments , text overlaps were revised accordingly

**Reviewers comments**

Reviewers comments were revised and responded one by one on the following way.

**Reviewer 1**

**Response :** No comments were provided by reviewer 1 and thank you for your time to review this manuscript.

**Reviewer 2**

Thank you so much for giving me the opportunity to review this study. Thanks the authors for their hard work with this manuscript.

1. In the title, the authors mentioned- “maternal malnutrition”. However, I see only anaemia was dealt with. Suggest restricting the title to anaemia instead of ‘malnutrition’ which is a broader term.

**Response :** First, authors would like to thank the reviewer for the constructive comments. The reason why the paper focused on maternal anemia is that maternal anemia is the most important contributor in maternal malnutrition. Among the indicators in maternal malnutrition, maternal anemia is common in Ethiopia due to parity and that was the reason we focused on anemia in the analysis section.

2. The introduction is quite large. More focus can be paid to methods (For even more clarity).

**Response :**The introduction was revised and based on the comments and the response on the method part was stated next in question number 3.

3. About the data source: The authors mentioned that- “The details on data sources and methods in Ethiopian subnational GBD analysis were reported on the health progress study in Ethiopia(30).” However, I feel it is quite relevant that you provide a brief summary of the data source, and how that data was collected. How the authors retrieved it and cleaned it. This is important as it is directly related to the outcome of interest. For example, I understand that stunting definition is <-2SD of height for age. But how the height and weight was measured? How did you calculate the standard deviation (The software). Did you exclude any outlier? Or, a readymade calculation was available and you used it directly. That kind of granularity should be available in the methods.

**Response :**

First, authors would like to thank the reviewer for the constructive comments. The estimates of malnutrition burden and the trends in its indicators for every regional state of the country were produced using the standardized GBD methods. The data source and how the data collected was clearly stated by the global burden of the disease estimation and how the data obtained was described in line 179-195. Yes, we used already readymade calculation and the general formula and calculations how it was done were stated on line 206-212

4. The authors used the term malnutrition synonymously with undernutrition, which is actually incorrect. Is there a reason why they are not using ‘undernutrition’ straightaway?

**Response :**

Thank you again for the critical comments. Sure, We used malnutrition instead of undernutrition and to avoid this confusion we primarily stated why we used this in the beginning of the introduction Line 107-108

5. Table 3: ‘Mothers’ or women of reproductive age? There are some typo errors in the table. Please revise

**Response : Comment was addressed on table 3**

6. The authors themselves expressed their concern about the source data in the limitation section. This is critical for the entire paper and I expressed my concern earlier. The authors must ensure adequate information about the data management and what kind of bias they expect, they should state clearly. The authors suggested in the same section what can be done in future. As a reviewer, I am concerned about the reliability of the present finding. Please focus there. Again, in the same place, they mentioned about what was not done. Please stick to what you have done**.**

**Response :**

Thank you for the comments again. W stated the problem of the quality of the data only for low birth weight analysis but not for the general analysis of the paper. This may allow for readers to carefully consider the result findings on low birth weight section. Of course we used all available data in the country for this analysis as stated in the method section. Other concerns were revised based on the given comments in line
